# Longitudinal remodeling of gastric microbiota following *Helicobacter pylori* eradication reveals an eradication-associated microbial signature in gastric cancer

**DOI:** 10.3389/fcimb.2026.1848437

**Published:** 2026-06-05

**Authors:** Chin-Hee Song, Yonghoon Choi, Nayoung Kim, Sungchan Ha, Ji Hyun Park, Ho-Kyoung Lee, Cheol Min Shin, Soyeon Ahn

**Affiliations:** 1 Department of Internal Medicine, Seoul National University Bundang Hospital, Seongnam, Republic of Korea; 2 Research Center for Sex- and Gender-Specific Medicine, Seoul National University Bundang Hospital, Seongnam, Republic of Korea; 3 Department of Internal Medicine and Liver Research Institute, Seoul National University College of Medicine, Seoul, Republic of Korea; 4 Medical Research Collaborating Center, Seoul National University Bundang Hospital, Seongnam, Republic of Korea

**Keywords:** follow-up studies, gastrointestinal microbiome, *Helicobacter pylori*, stomach neoplasms, treatment outcome

## Abstract

**Introduction:**

*Helicobacter pylori* (*H. pylori*) is a major cause of gastric cancer (GC); however, GC also develops in *H. pylori*–negative patients, and the characteristics of non-*H. pylori* microbial communities remain unclear.

**Methods:**

We characterized gastric microbiota in patients with GC according to *H. pylori* status, sex, GC subtype, and longitudinal changes following *H. pylori* eradication therapy. Gastric corpus mucosal samples were collected from 35 patients with GC who underwent endoscopic therapy and longitudinal follow-up. Some patients were followed for more than 10 years, and gastric microbiota were analyzed using 16S rRNA gene sequencing.

**Results:**

*H. pylori*–negative samples exhibited significantly higher microbial diversity and distinct community structures compared with *H. pylori*–positive samples, which was consistent across sex and GC subtypes. Multiple non-*H. pylori* taxa were enriched in *H. pylori*–negative samples, including organisms with reported urease and nitrate-reducing activities. Longitudinal analyses demonstrated that successful eradication induced significant but non-uniform microbial shifts, whereas persistent infection maintained stable *H. pylori*–dominated profiles. Notably, species-level analyses revealed selective and *H. pylori* eradication-specific microbial changes, with *Actinomyces naeslundii* consistently enriched only in the *H. pylori* eradicated group across longitudinal modeling and differential abundance analyses. Functional prediction analyses revealed a reduced representation of host–pathogen interaction–related pathways in *H. pylori*–negative samples.

**Discussion:**

These findings suggest that *H. pylori*–negative gastric microbiota harbor functionally distinct microbial communities that may contribute to gastric carcinogenesis through alternative microbial and ecological pathways. In addition, *H. pylori* eradication revealed a longitudinal remodeling of gastric microbiota.

## 1 Introduction

Gastric cancer (GC) remains a major global health burden, ranking among the most commonly diagnosed cancers, and the leading cause of cancer-related mortality worldwide (Bray et al., 2024).

This burden is particularly high in East Asia, including South Korea, largely reflecting the historically high prevalence of *Helicobacter pylori* (*H. pylori*) infection (Park et al., 2025). Although the incidence and mortality rates of GC have declined in recent decades, it remains a significant public health concern, particularly in aging populations (Morgan et al., 2022; Thrift et al., 2023). *H. pylori* infection is the primary risk factor for non-cardia GC and has been classified as a Group I carcinogen by the International Agency for Research on Cancer. Chronic infection of *H. pylori* induces persistent gastric inflammation and promotes a multistep carcinogenic process that progresses from gastritis to carcinoma (Correa, 1992; Polk and Peek, 2010). However, only a subset of infected individuals develops GC, suggesting that additional microbial, host, and environmental factors contribute to gastric carcinogenesis (Amieva and Peek, 2016).

Although eradication therapy reduces the risk of GC, it does not completely eliminate it (Sugano et al., 2015; Chen et al., 2016), raising the possibility that microbial factors other than *H. pylori* may play a role in tumorigenesis (Noto and Peek, 2017; Kim, 2024). Furthermore, GC is histologically heterogeneous and is classified into intestinal- and diffuse-type tumors according to the Lauren classification, which differ in their pathogenesis and clinical behavior (Lauren, 1965; Correa, 1992). These observations suggest that gastric carcinogenesis may involve complex interactions among *H. pylori*, the broader gastric microbiome, and host-related factors.

Advances in sequencing technologies and analytical approaches have enabled the detailed characterization of the human gastric microbiota (Bik et al., 2006; Noto and Peek, 2017). Despite the highly acidic environment of the stomach, various bacterial genera, including *Streptococcus*, *Prevotella*, *Veillonella*, *Rothia*, and *Neisseria*, have been identified in healthy individuals (Bik et al., 2006; Aviles-Jimenez et al., 2014). Studies have also suggested that the gastric microbial community undergoes changes during disease progression, with alterations in microbial diversity observed in gastritis, intestinal metaplasia, and GC (Coker et al., 2018; Ferreira et al., 2018). However, the relationship between gastric microbiota and gastric carcinogenesis remains incompletely understood, and findings across studies have been inconsistent (Noto and Peek, 2017; Ferreira et al., 2018).

Previously, we demonstrated that aging is associated with structural changes in the gastric mucosa and reduced gastric acid secretion, accompanied by decreased expression of H^+^–K^+^-ATPase in the stomach (Kang et al., 2010; Jo et al., 2013). We further revealed that mucosa-associated microbiota profiles differ between gastric regions and that the gastric corpus microbiota may be particularly informative in identifying non-*H. pylori* bacteria involved in gastric carcinogenesis (Jo et al., 2016; Sohn et al., 2017). In a prospective cohort study, gastric microbiota profiles differed according to *H. pylori* infection status and could be classified into distinct gastrotypes, with Akkermansia-dominant profiles linked to a higher risk of metachronous gastric neoplasms (Lee et al., 2024). In a longitudinal study, we also demonstrated that the gastric microbiota composition changes over time and following *H. pylori* eradication, with age-related declines in microbial diversity and heterogeneous microbial responses to *H. pylori* eradication therapy (Shin et al., 2020). However, most previous studies have been limited to cross-sectional analyses, and the role of bacterial species other than *H. pylori* in gastric carcinogenesis remains poorly understood. Given that GC exhibits heterogeneity according to *H. pylori* infection status, sex, and tissue type, we hypothesized that gastric bacterial communities would differ according to these factors. Therefore, this study aimed to characterize the gastric microbiota in patients with GC using next-generation sequencing, with a particular focus on identifying bacterial taxa associated with carcinogenesis in *H. pylori*–negative GCs and evaluating longitudinal microbiota changes following *H. pylori* eradication therapy.

## 2 Materials and methods

### 2.1 Study subjects

This study evaluated the longitudinal changes in the gastric corpus mucosa–associated microbiota in individuals without significant gastroduodenal disease. A total of 35 participants were included, all of whom underwent upper gastrointestinal endoscopy at baseline and at least one follow-up endoscopy more than one year after enrollment. Longitudinal gastric corpus mucosal biopsy samples were collected from each participant at a minimum of two time points, including baseline and follow-up surveillance endoscopies. Detailed longitudinal sampling information, including the number of samples per patient and follow-up duration, is provided in **Supplementary Table 1**. All enrolled patients were diagnosed as gastric cancer histologically before and after endoscopic submucosal dissection. All participants underwent longitudinal clinical follow-up with repeated endoscopic examinations, including two individuals who were followed for more than 10 years. The participants were categorized according to their *H. pylori* status, sex, and histological subtype of GC based on the Lauren classification (intestinal type vs. diffuse type). Participants were recruited from Seoul National University Bundang Hospital between August 16, 2005, and October 11, 2018. All participants were of Korean origin. Upper gastrointestinal endoscopy was performed primarily as part of a screening program for premalignant gastric mucosal lesions or GC, or for the evaluation of mild dyspeptic symptoms. GC was diagnosed based on histopathological examination of specimens obtained by endoscopic submucosal dissection. To minimize potential confounding effects on the gastric microbiota, individuals with a history of proton pump inhibitor, histamine-2 receptor antagonist, or non-steroidal anti-inflammatory drug use at baseline or follow-up were excluded. Participants who had received antibiotics within four weeks prior to sample collection, had undergone gastric surgery, had a history of *H. pylori* eradication therapy, or had systemic diseases requiring long-term medication were also excluded.

All endoscopic procedures were performed by a single experienced gastroenterologist (N.K.), and 10 gastric biopsy specimens were obtained from each participant according to a standardized protocol. Prior to endoscopy, participants completed a structured questionnaire under the supervision of a trained interviewer. The questionnaire assessed demographic characteristics, family history of GC in first-degree relatives, smoking status, alcohol consumption, antibiotic exposure, and history of *H. pylori* treatment. To prevent cross-contamination, the endoscopes were thoroughly cleaned and disinfected using a detergent solution containing proteolytic enzymes and glutaraldehyde, and sterilized biopsy forceps were used for each sampling procedure. Biopsy specimens were immediately stored at −80 °C until further analysis. *H. pylori* infection status was initially assessed using conventional diagnostic methods, including the urea breath test, rapid urease test, serology, histological examination, and bacterial culture. Subjects with at least one positive result among these tests were considered to have *H. pylori* infection. For microbiome-based analyses and final subgroup classification, *H. pylori* status was subsequently defined according to next-generation sequencing results, with subjects classified as *H. pylori*–positive if the relative abundance of *H. pylori* sequences was ≥1%, a threshold previously proposed to reflect gastric colonization (Kim et al., 2015). Using this sequencing-based threshold at baseline, four individuals initially classified as *H. pylori*–positive by conventional diagnostic methods were classified as *H. pylori*–negative based on sequencing results, whereas one individual initially classified as *H. pylori*–negative was classified as *H. pylori*–positive by sequencing analysis. The study protocol was reviewed and approved by the Institutional Review Board of Seoul National University Bundang Hospital (approval no. B-1903-529-302). All research procedures involving human subjects were conducted in compliance with the internationally accepted ethics principles, including those outlined in the Declaration of Helsinki and its subsequent revisions. Written informed consent was obtained from all participants prior to their participation in the study.

### 2.2 *H. pylori* testing and histological assessment

During each endoscopic examination, 10 gastric biopsy specimens were obtained from standardized sites in the antrum and corpus, including both the greater and lesser curvatures, to assess current *H. pylori* infection and histopathological features of the gastric mucosa (Shin et al., 2010). Histological evaluation was performed using four biopsy specimens collected from the greater curvature of the antrum and corpus. The tissues were fixed in formalin and stained with hematoxylin and eosin to assess inflammatory cell infiltration, glandular atrophy, and intestinal metaplasia. The presence of *H. pylori* was examined using modified Giemsa staining. Histological findings were graded according to the updated Sydney system (0: none; 1: mild; 2: moderate; 3: marked) (Dixon et al., 1996). Rapid urease testing was conducted using biopsy specimens obtained from the lesser curvature of the antrum and corpus (*Campylobacter*-like organism test [CLO test]; DeltaWest, Bentley, Australia). For bacterial culture, two additional biopsy specimens, one from the antrum and the other from the corpus, were processed under microaerophilic conditions. The isolated organisms were identified as *H. pylori* using Gram staining, colony morphology, and positive oxidase, catalase, and urease results. Current *H. pylori* infection was defined as a positive result in at least one of the following biopsy-based tests: histological examination, CLO test, or culture. Remaining biopsy specimens and GC tissues were immediately stored at −80 °C until DNA extraction.

### 2.3 *H. pylori* serology and evaluation of gastric atrophy

Fasting blood samples were collected from all participants at baseline. To differentiate past from current infections, serum *H. pylori*–specific immunoglobulin G (IgG) levels were measured using an enzyme-linked immunosorbent assay (Genedia *H. pylori* ELISA; Green Cross Medical Science Corp., Eumseong, South Korea) (Yoon et al., 2011), which employs antigens derived from Korean *H. pylori* strains (Kim et al., 2014). Serum concentrations of pepsinogen (PG) I and II were measured using a latex-enhanced turbidimetric immunoassay (Shima Laboratories, Tokyo, Japan) to evaluate the extent of gastric mucosal atrophy. Gastric atrophy was defined as a PG I/II ratio < 2.5, whereas the absence of atrophy was defined as a PG I level > 70 ng/mL and a PG I/II ratio > 4.0.

### 2.4 DNA preparation

Genomic DNA was isolated from non-neoplastic gastric corpus biopsy specimens as described previously (Shin et al., 2020). Briefly, tissue samples were mechanically disrupted in a proteinase K–containing lysis buffer (20 mmol/L Tris–HCl, pH 8.0; 10 mmol/L EDTA; 0.5% sodium dodecyl sulfate; proteinase K) using a sterile micropestle, followed by enzymatic digestion at 52 °C for 3 h. After complete lysis, the DNA was purified using a phenol–chloroform extraction procedure and subsequently precipitated with ethanol. Purified DNA was collected and stored for downstream sequencing.

### 2.5 Polymerase chain reaction amplification and illumina sequencing

The V3–V4 hypervariable regions of the bacterial 16S rRNA gene were amplified by PCR using the primers 341F (5’-TCGTCGGCAGCGTCAGATGTGTATAAGAGACAGCCTACGGGNGGCWGCAG-3’) and 805R (5’-GTCTCGTGGGCTCGGAGATGTGTATAAGAGACAGGACTACHVGGGTATCTAATCC-3’). Primary amplification was performed with an initial denaturation at 95 °C for 3 min, followed by 25 cycles of 95 °C for 30 s, 55 °C for 30 s, and 72 °C for 30 s and a final extension at 72 °C for 5 min.

Indexed sequencing adapters were subsequently incorporated through a second PCR using Nextera i5 (5′-AATGATACGGCGACCACCGAGATCTACACXXXXXXXX-TCGTCGGCAGCGTC-3′) and i7 (5′-CAAGCAGAAGACGGCATACGAGATXXXXXXXX-AGTCTCGTGGGCTCGG-3′) primers, where “X” denotes the barcode sequence. The indexing reaction was conducted under identical thermal conditions, except with the number of amplification cycles reduced to eight.

Amplicon size was verified by 1% agarose gel electrophoresis and visualized using a Gel Doc system (Bio-Rad, Hercules, CA, USA). PCR products were purified using a QIAquick PCR Purification Kit (Qiagen, Valencia, CA, USA), quantified, and pooled at equimolar concentrations. Short non-specific fragments were removed using AMPure beads (Agencourt Bioscience, MA, USA). Library integrity and fragment distribution were assessed using Bioanalyzer 2100 (Agilent, Palo Alto, CA, USA) equipped with a DNA 7500 chip. The final pooled libraries were sequenced on an Illumina MiSeq platform (Illumina, USA) at CJ Bioscience, Inc. (Seoul, Korea) according to the manufacturer’s protocol.

### 2.6 Sequencing data processing

Raw sequencing reads were subjected to quality control prior to downstream analysis. Low-quality reads (Phred score < 25) were removed using Trimmomatic v0.32 (Bolger et al., 2014). Paired-end reads were assembled using PANDA-seq (Masella et al., 2012). Primer sequences were trimmed using an in-house pipeline provided by CJ Bioscience (Seoul, South Korea) with a similarity threshold of 0.8. Non-specific amplicons that did not correspond to bacterial 16S rRNA genes were filtered using hmmsearch in the HMMER package (Eddy, 2011) based on 16S rRNA Hidden Markov Models. Sequencing errors were corrected using DUDE-Seq (Lee et al., 2017), and redundant sequences were collapsed using UCLUST (Edgar, 2010). Chimeric sequences were identified and removed using UCHIME (Edgar et al., 2011) against the EzBioCloud non-chimeric 16S rRNA reference database. Reads with <97% similarity to the reference database were excluded from analysis. The remaining high-quality sequences were clustered into operational taxonomic units (OTUs) with 97% sequence similarity using CD-HIT (Fu et al., 2012) and UCLUST (Edgar, 2010). Taxonomic assignment was performed against the EzBioCloud database using USEARCH v8.1.1861_i86linux32 (Edgar, 2010).

### 2.7 Statistical analysis and visualization

Microbial community analyses were performed using the EzBioCloud Microbiome Analysis Platform (CJ Biosciences). To minimize bias due to sequencing depth, reads were rarefied to 11,729 reads per sample prior to diversity analyses. Alpha diversity indices, including observed operational taxonomic units (OTUs), Chao1 richness, and Shannon diversity indices, were calculated based on the OTU table. Beta diversity was assessed using generalized UniFrac distances, and differences in community structure among groups were visualized using principal coordinate analysis (PCoA). The statistical significance of group separation was evaluated using permutational multivariate analysis of variance (PERMANOVA).

Relative taxonomic abundance (%) was used to identify discriminatory taxa between groups using linear discriminant analysis effect size (LEfSe) (Segata et al., 2011) at the species levels. All statistical analyses, except those related to sequencing data processing, were performed using PASW Statistics version 18.0.0 (SPSS Inc., Chicago, IL, USA). Multiple-group comparisons were conducted using the Kruskal–Wallis H test, followed by pairwise comparisons using the Mann–Whitney U test when appropriate.

For longitudinal analyses, linear mixed-effects models were applied to assess temporal changes in microbial taxa, while accounting for repeated measurements within individuals. Microbial relative abundances (%) were used as outcome variables and were log-transformed when appropriate to approximate a normal distribution. Follow-up time was treated as a continuous variable. Fixed variables included follow-up time, study group, and their interaction, whereas participant identification was included as a random intercept to account for within-individual correlations. The interaction term (time × group) was used to evaluate whether the temporal trajectories differed between groups. The interaction model was interpreted when the interaction term was statistically significant; otherwise, an additive model without the interaction term was used. Model parameters were estimated using restricted maximum likelihood (REML), and statistical significance was assessed using Satterthwaite’s approximation for the degrees of freedom. Pairwise comparisons between groups were performed using estimated marginal means (emmeans) with Tukey-adjusted *P*-values. Model-based estimated marginal means were used to visualize longitudinal trajectories (predicted trajectory plots), and individual-level changes were illustrated using spaghetti plots. In some models, convergence warnings and singular fit conditions were observed (e.g., boundary (singular) fit with gradient ≈ 0), indicating that the variance of the random intercept was estimated to be close to zero. In these cases, the random-effects structure contributed minimally to the model. Therefore, fixed-effects estimates were retained for interpretation, whereas random effects were interpreted with caution. Such situations are not uncommon in longitudinal microbiome data with limited between-subject variability.

A *p*-value < 0.05 was considered statistically significant. False discovery rate (FDR) correction was applied to adjust for multiple testing, and *q*-values < 0.05 were considered significant after correction.

Taxonomic compositions were visualized as bar plots showing relative abundances at the phylum level using GraphPad Prism (version 5.01, GraphPad Software, San Diego, CA, USA). PCoA and LEfSe LDA score bar plots were formatted using Microsoft Excel (Microsoft Corp., Redmond, WA, USA) for standardized graphical presentation. The relative abundances across phyla, classes, orders, families, genera, and species levels are provided in **Supplementary Datasheet S1**.

## 3 Results

### 3.1 Study design and analytical framework

**Figure 1:**
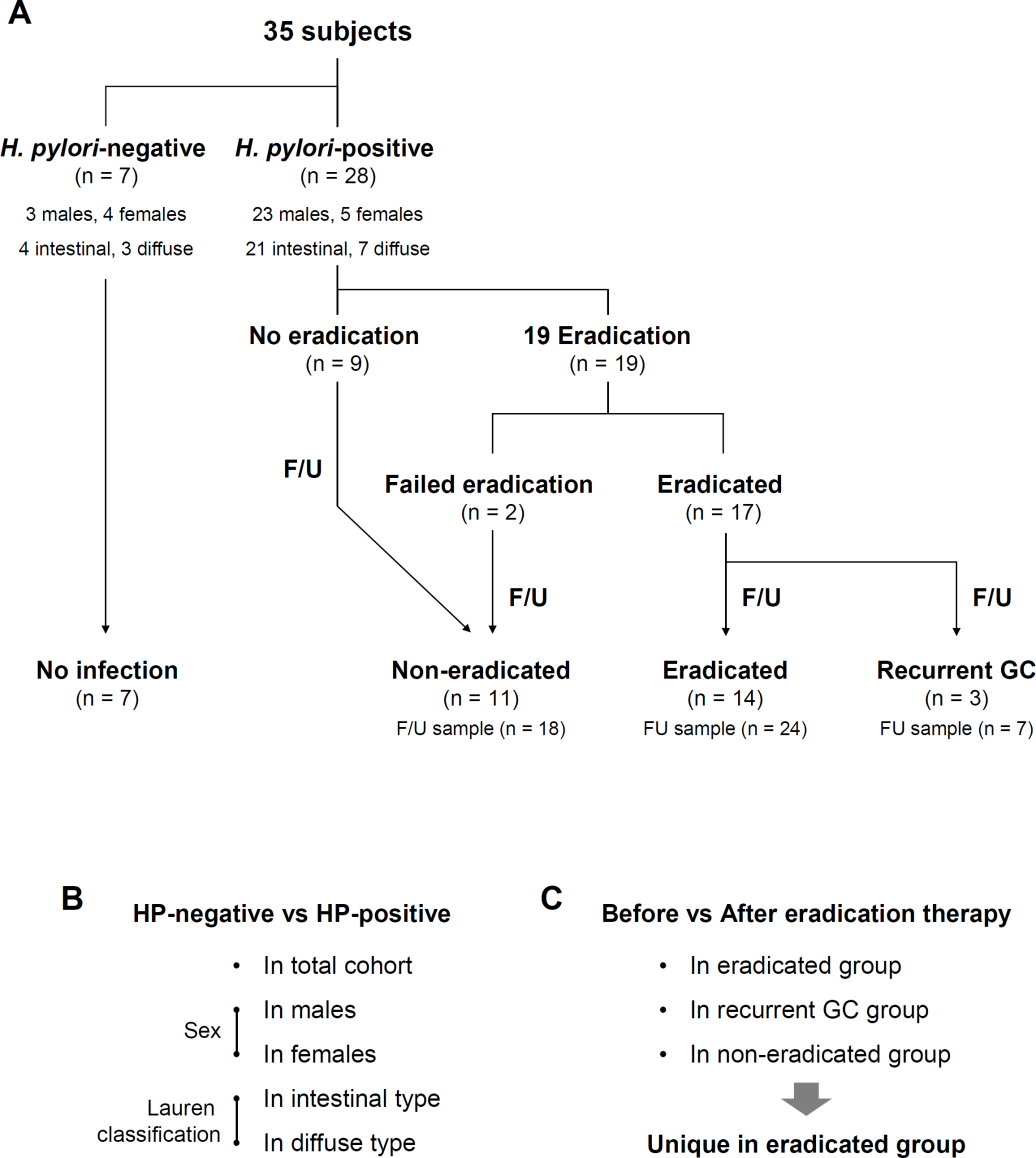
Study design and analytical framework. **(A)** Classification of participants according to *H. pylori* infection status and longitudinal eradication outcomes. Seven subjects were classified as *H. pylori*–negative (no-infection group). *H. pylori*–positive patients were further categorized into three outcome-based groups: eradicated (n = 14), recurrent GC after successful eradication (n = 3), and non-eradicated (n = 11), the latter comprising eradication-naive (n = 9) and eradication-failure (n = 2) subgroups. **(B)** Cross-sectional comparison framework according to *H. pylori* status in the total cohort, with additional stratification by sex and GC subtype based on the Lauren classification (intestinal vs. diffuse type). **(C)** Longitudinal comparison of microbial profiles within *H. pylori*–positive patients, including before- and after-eradication analyses across outcome groups and assessment of temporal changes during follow-up. This framework enabled the evaluation of group-specific microbial dynamics and the identification of taxa associated with successful eradication.

The overall study design and analytical framework are illustrated in **Figure 1**. Participants were categorized according to *H. pylori* status, sex, and gastric cancer subtype based on the Lauren classification. Among the total cohort (n = 35), seven participants were classified as *H. pylori*–negative and defined as the no-infection group. Among *H. pylori*–positive patients (n = 28), longitudinal follow-up enabled classification into three outcome-based groups: an eradicated group (n = 14), a recurrent GC group following successful eradication (n = 3), and a non-eradicated group (n = 11), which comprised both eradication-naive patients (n = 9) and those with eradication failure (n = 2) (**Figure 1A**). This grouping reflects distinct clinical trajectories following *H. pylori* infection and treatment. Comparative analyses were first conducted across *H. pylori* status in the total cohort and further stratified by sex and histological subtype (**Figure 1B**). For longitudinal analyses, within *H. pylori*–positive patients, microbial profiles were compared before and after eradication therapy across the three outcome groups, and temporal dynamics were assessed according to follow-up duration (**Figure 1C**). This design revealed distinct group-specific longitudinal changes in microbial composition across eradication outcomes, enabling the identification of taxa associated with successful eradication.

### 3.2 Global alterations in gastric microbiota according to *H. pylori* infection in the total cohort

The baseline characteristics of the study participants are summarized in **Table 1**. There were no significant differences between the groups in sociodemographic variables, including sex, age, body mass index (BMI), alcohol consumption, smoking status, family history of GC in first-degree relatives, and endoscopic and histologic gastritis.

**Table 1:** Baseline characteristics.

Variables			Total (N = 28) (%)	Non-eradicated (n=11) (%)	Eradicated		
					Without metachronous recurrence (n=14) (%)	Metachronous recurrence (n=3) (%)	*P*-value
Sex		Male	23 (82.1)	10 (90.9)	10 (71.4)	3 (100.0)	0.313
		Female	5 (17.9)	1 (9.1)	4 (28.6)	0 (0.0)	
Age			61.61 ± 12.90	68.64 ± 11.16	56.86 ± 12.84	58.00 ± 10.15	0.061
BMI			23.60 ± 4.63	22.92 ± 3.86	23.59 ± 5.53	26.09 ± 2.07	0.594
Alcohol		None	10 (35.7)	5 (45.5)	5 (35.7)	0 (0.0)	0.713
		Ex	6 (21.4)	2 (18.2)	3 (21.4)	1 (33.3)	
		Current	12 (42.9)	4 (36.4)	6 (42.9)	2 (66.7)	
Smoking		None	11 (39.3)	4 (36.4)	6 (42.9)	1 (33.3)	0.696
		Ex	11 (39.3)	5 (45.5)	4 (28.6)	2 (66.7)	
		Current	6 (21.4)	2 (18.2)	4 (28.6)	0 (0.0)	
Family history of GC		No	25 (89.3)	11 (100.0)	12 (85.7)	2 (66.7)	0.211
		Yes	3 (10.7)	0 (0.0)	2 (14.3)	1 (33.3)	
Presence of Gastritis	Endoscopic AG	No	9 (32.1)	2 (18.2)	7 (50.0)	0 (0.0)	0.329
		Closed	11 (39.3)	5 (45.5)	4 (28.6)	2 (66.7)	
		Open	8 (28.6)	4 (36.4)	3 (21.4)	1 (33.3)	
	Endoscopic IM	No	11 (39.3)	2 (18.2)	8 (57.1)	1 (33.3)	0.137
		Yes	17 (60.7)	9 (81.8)	6 (42.9)	2 (66.7)	
	Histologic AG	No	12 (42.9)	2 (18.2)	8 (57.1)	2 (66.7)	0.100
		Yes	16 (57.1)	9 (81.8)	6 (42.9)	1 (33.3)	
	Histologic IM	No	8 (28.6)	2 (18.2)	6 (42.9)	0 (0.0)	0.204
		Yes	20 (71.4)	9 (81.8)	8 (57.1)	3 (100.0)	
	PG I/II ratio		2.41 ± 1.10	1.96 ± 0.89	2.82 ± 1.24	2.27 ± 0.49	0.161
HP status	Rapid urease test	Negative	7 (25.9)	2 (20.0)	4 (28.6)	1 (33.3)	0.852
		Positive	20 (74.1)	8 (80.0)	10 (71.4)	2 (66.7)	
	Histology	Negative	6 (21.4)	3 (27.3)	3 (21.4)	0 (0.0)	0.594
		Positive	22 (78.6)	8 (72.7)	11 (78.6)	3 (100.0)	
	HpIgG	Negative	4 (14.3)	2 (18.2)	2 (14.3)	0 (0.0)	0.727
		Positive	24 (85.7)	9 (81.8)	12 (85.7)	3 (100.0)	
	Culture	Negative	9 (45.0)	4 (50.0)	4 (44.4)	1 (33.3)	0.884
		Positive	11 (55.0)	4 (50.0)	5 (55.6)	2 (66.7)	
Characteristics of initial of GC	Stage	EGC	26 (92.9)	10 (90.9)	13 (92.9)	3 (100.0)	0.863
		AGC	2 (7.1)	1 (9.1)	1 (7.1)	0 (0.0)	
	Location	Upper third	1 (3.6)	1 (9.1)	0 (0.0)	0 (0.0)	0.110
		Middle third	8 (28.6)	1 (9.1)	7 (50.0)	0 (0.0)	
		Lower third	19 (67.8)	9 (81.8)	7 (50.0)	3 (100.0)	
	Histology	Intestinal	20 (71.4)	10 (90.9)	7 (50.0)	3 (100.0)	0.041
		Diffuse	8 (28.6)	1 (9.1)	7 (50.0)	0 (0.0)	
	Initial treatment	Endoscopic resection	17 (60.7)	7 (63.6)	7 (50.0)	3 (100.0)	0.265
		Surgery	11 (39.3)	4 (36.4)	7 (50.0)	0 (0.0)	

BMI, body mass index; GC, gastric cancer; AG, atrophic gastritis; IM, intestinal metaplasia; HP, *Helicobacter pylori*; PG, pepsinogen; HpIgG; *H. pylori* immunoglobulin G; EGC, early gastric cancer; AGC, advanced gastric cancer.

**Figure 2:**
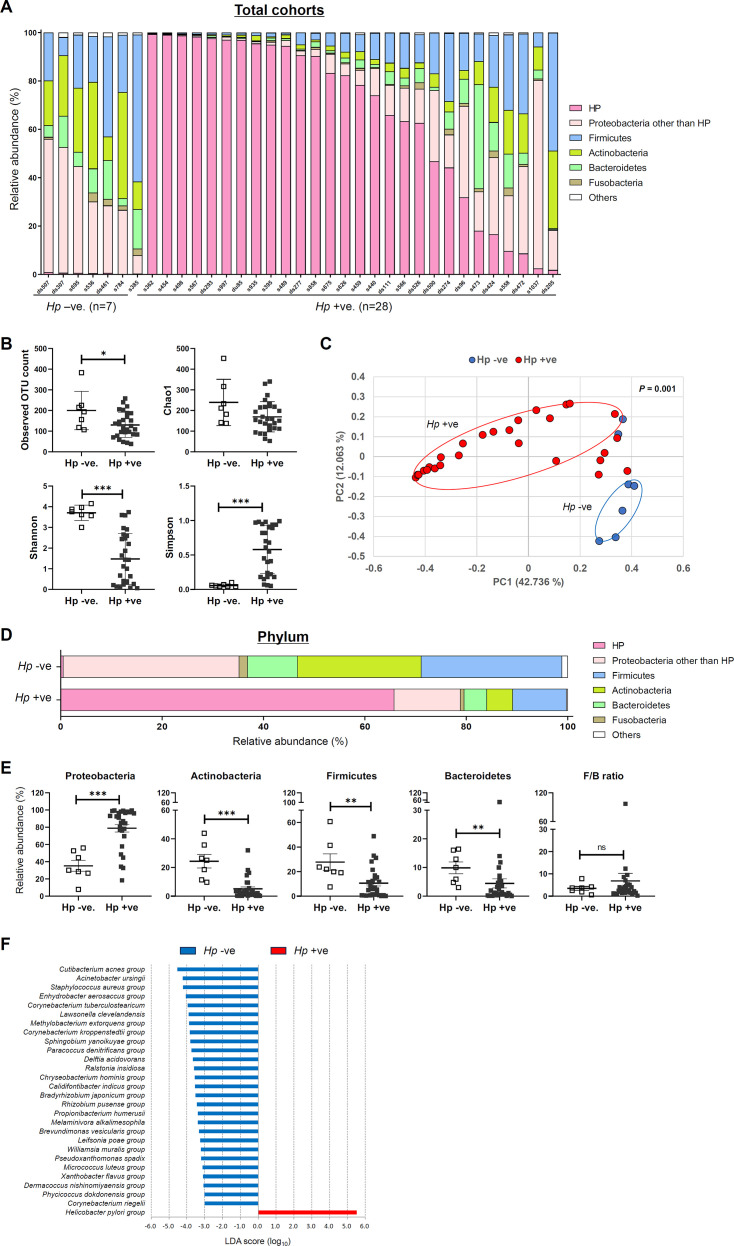
Global alterations in gastric microbiota according to *H. pylori* infection in the total cohort. **(A)** Relative abundance of bacterial taxa at the phylum level in *H. pylori*–negative and *H. pylori*–positive samples. **(B)** Alpha diversity indices, including observed OTUs, Chao1 richness estimator, Shannon diversity index, and Simpson index. **(C)** PCoA plot based on generalized UniFrac distances showing separation between *H. pylori*–negative and *H. pylori*–positive samples. **(D)** Relative abundance of major bacterial phyla. **(E)** Relative abundance of major phyla and F/B ratio in total cohorts according to *H. pylori* status. **(F)** LEfSe identifying differentially abundant taxa between *H. pylori*–negative and *H. pylori*–positive samples. Only taxa with defined species-level annotation and LDA scores above the predefined threshold (≥3-fold) are shown. Blue bars indicate taxa enriched in *H. pylori*–negative, and red bars indicate taxa enriched in *H. pylori*–positive samples. Statistical significance was assessed using the Kruskal–Wallis test followed by the Mann–Whitney U test where appropriate. Differences in beta diversity were evaluated using PERMANOVA. *, *P* < 0.05; **, *P* < 0.01; ***, *P* < 0.001; n.s., not significant for two-group comparison. OTU, operational taxonomic unit; PCoA, principal coordinate analysis; UniFrac, phylogenetic distance metric; LEfSe, linear discriminant analysis effect size; LDA, linear discriminant analysis; F/B ratio, Firmicutes to Bacteroidetes ratio; PERMANOVA, permutational multivariate analysis of variance.

In the total cohort, *H. pylori*–negative samples exhibited a more diverse and evenly distributed microbial community compared with *H. pylori*–positive samples, as illustrated by the phylum-level relative abundance plots (**Figure 2A**). In contrast, *H. pylori*–positive samples were characterized by a predominance of *H. pylori*, reflecting a compositional shift toward a single dominant taxon. Consistent with this pattern, alpha diversity was significantly higher in *H. pylori*–negative samples, as evidenced by the increased Shannon diversity and decreased Simpson index (**Figure 2B**). The observed OTU count was also significantly higher in the *H. pylori*–negative group, whereas the Chao1 richness did not differ significantly between the groups, suggesting that the differences were primarily driven by community evenness rather than estimated richness. Beta diversity analysis based on generalized UniFrac distances demonstrated a clear separation between *H. pylori*–negative and *H. pylori*–positive samples (**Figure 2C**), indicating that *H. pylori* infection was associated with substantial restructuring of the gastric microbial community.

At the phylum level, *H. pylori*–negative samples showed significantly higher relative abundances of Actinobacteria, Firmicutes, and Bacteroidetes, whereas Proteobacteria was significantly enriched in *H. pylori*–positive samples (**Figures 2D, E**). The Firmicutes/Bacteroidetes ratio did not differ significantly between groups (**Figure 2E**).

LEfSe analysis further revealed that multiple non-*Helicobacter* taxa were enriched in the *H. pylori*–negative group, including *Cutibacterium acnes*, *Acinetobacter ursingii*, *Staphylococcus aureus*, and several *Corynebacterium*-related taxa (**Figure 2F**). Notably, several of these taxa have been reported to possess urease activity or nitrate-reducing capacity, suggesting their potential involvement in alternative urease-producing and nitrosating microbial pathways in the absence of *H. pylori*. In contrast, *H. pylori* was the primary taxon in the *H. pylori*–positive group.

### 3.3 Sex-specific differences in gastric microbial alterations according to *H. pylori* infection

**Figure 3:**
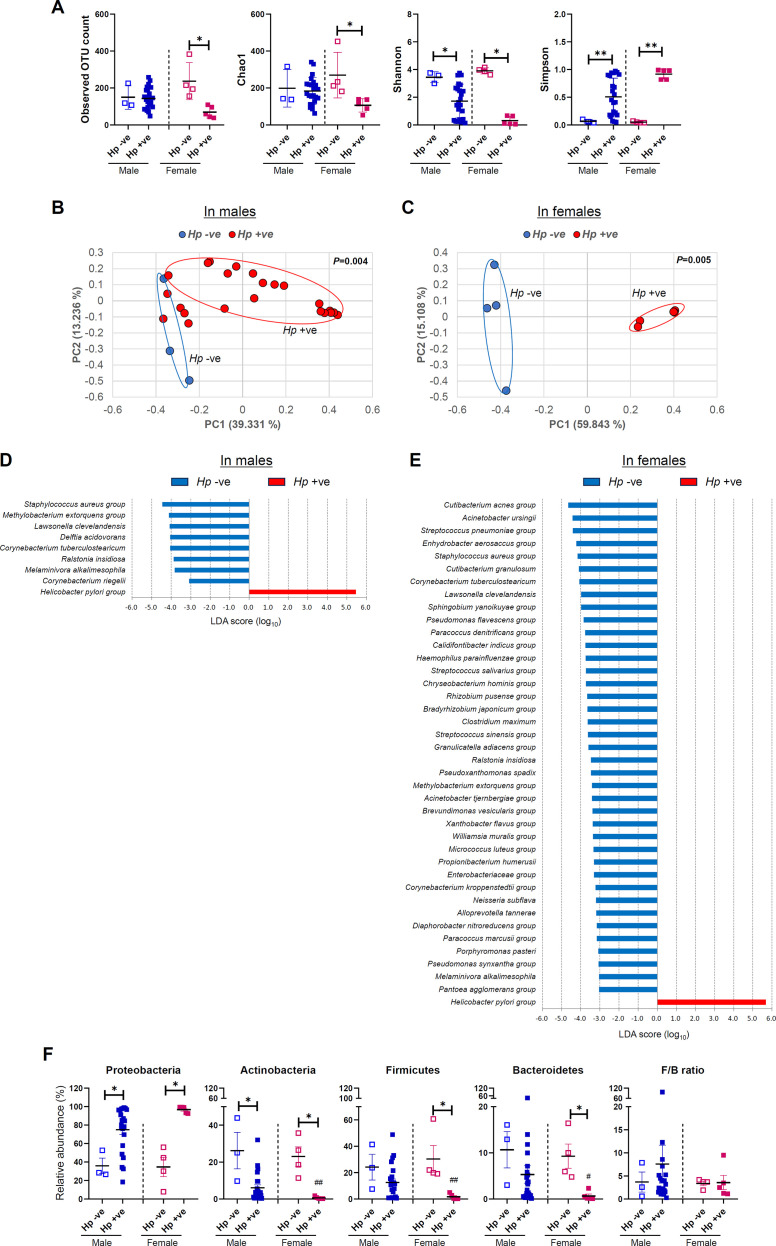
Sex-stratified differences in gastric microbiota according to *H. pylori* infection. **(A)** Alpha diversity indices (observed OTUs, Chao1, Shannon, and Simpson) in male and female groups stratified by *H. pylori* infection status. **(B)** PCoA plot for males based on generalized UniFrac distances. **(C)** PCoA plot for females. **(D, E)** LEfSe analysis identifying differentially abundant taxa between *H. pylori*–negative and *H. pylori*–positive samples in males **(D)** and females **(E)**. Only taxa with defined species-level annotation and LDA scores above the predefined threshold (≥3-fold) are shown. Blue bars indicate taxa enriched in *H. pylori*–negative, and red bars indicate taxa enriched in *H. pylori*–positive samples. **(F)** Relative abundance of major phyla and F/B ratio in males and females according to *H. pylori* status. Statistical analyses, including PERMANOVA for beta diversity, were performed as described in **Figure 2**. *, *P* < 0.05; **, *P* < 0.01 for two-group comparison. OTU, operational taxonomic unit; PCoA, principal coordinate analysis; LEfSe, linear discriminant analysis effect size; LDA, linear discriminant analysis; F/B ratio, Firmicutes to Bacteroidetes ratio.

When the cohort was stratified by sex, *H. pylori*–negative samples consistently exhibited greater microbial diversity and a broader range of taxa than *H. pylori*–positive samples in both males and females (**Figure 3**).

In males, alpha diversity patterns indicated reduced diversity in *H. pylori*–positive samples, as reflected by decreased Shannon diversity and increased Simpson index, while the observed OTU count and Chao1 richness did not differ significantly between the groups (**Figure 3A**). In contrast, in females, *H. pylori*–negative samples showed higher observed OTU counts and Chao1 richness, together with higher Shannon diversity and a lower Simpson index than *H. pylori*–positive samples (**Figure 3A**), indicating that both richness and diversity components were preserved in the absence of *H. pylori*. Beta diversity analysis demonstrated a clear separation between *H. pylori*–negative and *H. pylori*–positive samples in both males (*P* = 0.004) and females (*P* = 0.005) (**Figures 3B, C**), indicating distinct microbial community structures according to *H. pylori* status across both sexes.

LEfSe analysis revealed that multiple non-*Helicobacter* taxa were enriched in *H. pylori*–negative samples in a sex-specific manner. In males, taxa such as *Staphylococcus aureus*, *Methylobacterium extorquens*, *Lawsonella clevelandensis*, *Delftia acidovorans*, and *Ralstonia insidiosa* were enriched (**Figure 3D**). In females, a broader range of taxa was enriched in *H. pylori*–negative samples, including *Cutibacterium acnes*, *Acinetobacter ursingii*, *Streptococcus pneumoniae*, *Enhydrobacter aerosaccus*, *Corynebacterium tuberculostearicum*, and *Neisseria subflava* (**Figure 3E**). *H. pylori* remained the primary discriminative taxon in the *H. pylori*–positive group in both sexes.

At the phylum level, *H. pylori*–negative samples exhibited a distinct microbial profile characterized by a lower relative abundance of *Proteobacteria* and a higher abundance of *Actinobacteria* in both males and females (**Figure 3F**). Sex-specific differences were observed in the *H. pylori*–negative group. In females, *H. pylori*–negative samples showed higher relative abundances of Firmicutes and Bacteroidetes than *H. pylori*–positive samples, whereas these differences were not observed in males, indicating that phylum-level alterations associated with *H. pylori* status were more extensive in females. The Firmicutes/Bacteroidetes ratio did not differ significantly according to *H. pylori* status in either sex (**Figure 3F**).

### 3.4 *H. pylori*–associated microbial alterations within GC subtypes

**Figure 4:**
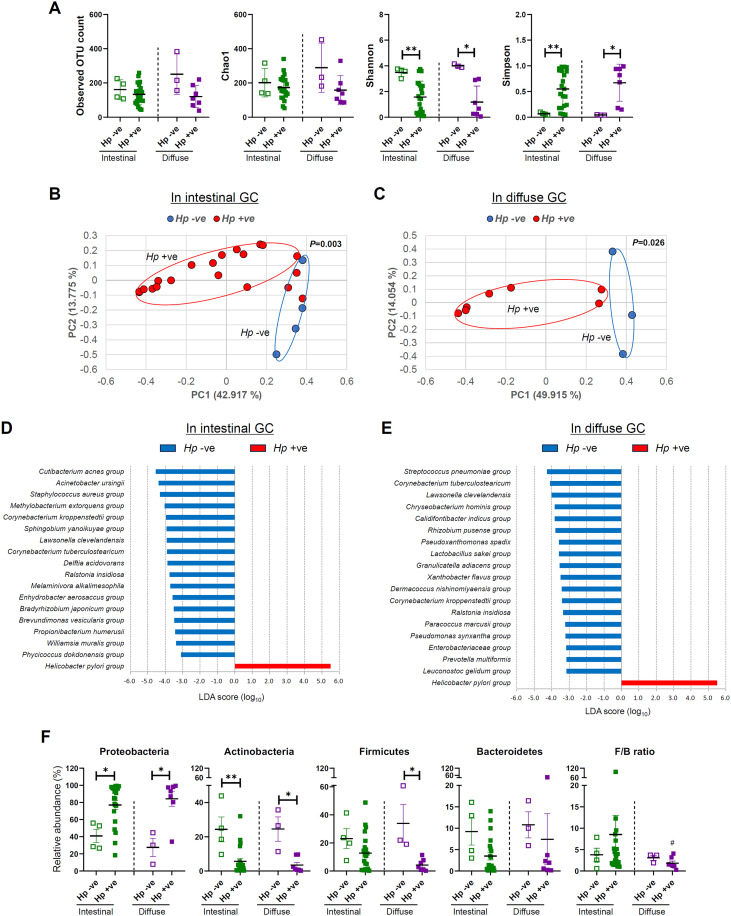
Gastric microbiota alterations within gastric cancer subtypes according to *H. pylori* infection. **(A)** Alpha diversity indices (observed OTUs, Chao1, Shannon, and Simpson) in intestinal-type and diffuse-type GC stratified by *H. pylori* infection status. **(B, C)** PCoA plot for intestinal-type **(B)** and diffuse-type gastric cancer **(C)**. **(D, E)** LEfSe analysis identifying differentially abundant taxa in intestinal-type **(D)** and diffuse-type GC **(E)**. Only taxa with defined species-level annotation and LDA scores above the predefined threshold (≥3-fold) are shown. Blue bars indicate taxa enriched in *H. pylori*–negative, and red bars indicate taxa enriched in *H. pylori*–positive samples. **(F)** Relative abundance of major phyla and F/B ratio according to *H. pylori* status within each GC subtype. Statistical analyses were performed as described in **Figure 2**. *, *P* < 0.05; **, *P* < 0.01 for two-group comparison. OTU, operational taxonomic unit; PCoA, principal coordinate analysis; LEfSe, linear discriminant analysis effect size; LDA, linear discriminant analysis; F/B ratio, Firmicutes to Bacteroidetes ratio; GC, gastric cancer.

To determine whether the gastric microbial profiles differed according to the Lauren classification in the absence of *H. pylori*, analyses were performed separately for intestinal- and diffuse-type GC (**Figure 4**).

In both intestinal- and diffuse-type GC, *H. pylori*–negative samples exhibited higher microbial diversity than *H. pylori*–positive samples, as reflected by increased Shannon diversity and decreased Simpson index (**Figure 4A**), while the observed OTUs and Chao1 richness did not differ significantly between groups. Beta diversity analysis demonstrated a clear separation between *H. pylori*–negative and *H. pylori*–positive samples within both intestinal- (**Figure 4B**; *P* = 0.003) and diffuse-type GC (**Figure 4C**; *P* = 0.026), indicating distinct microbial community structures according to *H. pylori* status across both subtypes.

LEfSe analysis revealed that *H. pylori*–negative samples harbored distinct microbial communities in a subtype-specific manner. In intestinal-type GC, *H. pylori*–negative samples were enriched with taxa such as *Cutibacterium acnes*, *Acinetobacter ursingii*, *Staphylococcus aureus*, *Methylobacterium extorquens*, *Corynebacterium kroppenstedtii*, *Lawsonella clevelandensis*, and *Ralstonia insidiosa* (**Figure 4D**). In diffuse-type GC, *H. pylori*–negative samples were enriched with a different set of taxa, including *Streptococcus pneumoniae*, *Corynebacterium tuberculostearicum*, *Lawsonella clevelandensis*, *Chryseobacterium hominis*, *Rhizobium pusense*, *Prevotella multiformis*, and *Leuconostoc gelidum* (**Figure 4E**). *H. pylori* remained the primary discriminative taxon in *H. pylori*–positive samples in both subtypes.

At the phylum level, *H. pylori*–negative samples consistently exhibited a lower relative abundance of Proteobacteria and higher abundance of Actinobacteria in both intestinal- and diffuse-type GC (**Figure 4F**). In addition, in diffuse-type GC, *H. pylori*–negative samples showed a higher relative abundance of Firmicutes than *H. pylori*–positive samples, whereas no significant difference was observed in intestinal-type GC. No significant differences in Bacteroidetes were observed according to *H. pylori* status in either subtype. The Firmicutes/Bacteroidetes ratio did not differ significantly between *H. pylori*–negative and *H. pylori*–positive samples in either subtype.

### 3.5 Longitudinal restructuring of gastric microbiota according to *H. pylori* eradication status in baseline *H. pylori*–positive subjects

**Figure 5:**
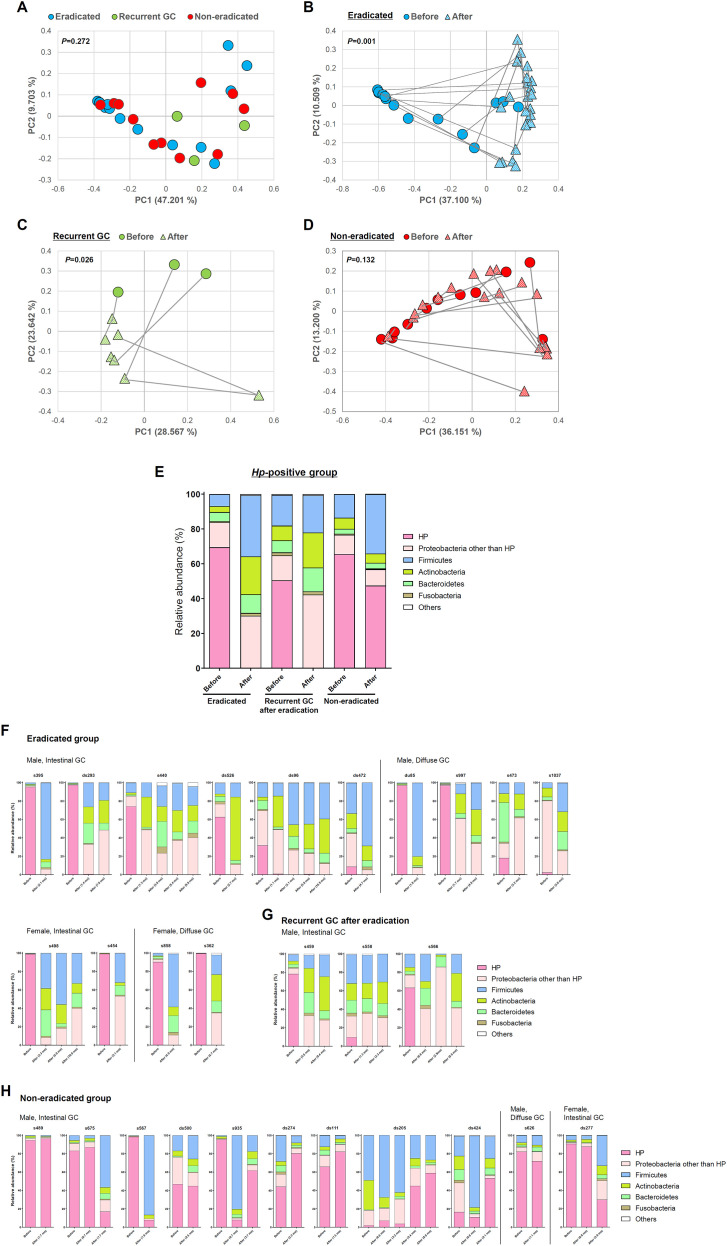
Longitudinal changes in gastric microbiota according to eradication status. **(A)** PCoA plot showing distribution of samples from eradicated, recurrent GC after eradication, and non-eradicated groups. **(B–D)** Trajectory plot of paired samples in the eradicated **(B)**, recurrent GC after eradication **(C)**, and non-eradicated group **(D)**. Lines indicate the direction of temporal change for each individual between baseline and follow-up. **(E)** Phylum-level relative abundance across the three groups. **(F–H)** Individual-level phylum composition in the eradicated **(F)**, recurrent GC after eradication **(G)**, and non-eradicated group **(H)**. PCoA, principal coordinate analysis; GC, gastric cancer.

Subsequent longitudinal analyses were restricted to subjects who were *H. pylori*-positive at baseline and were classified into three groups according to follow-up outcomes: eradicated, recurrent GC after eradication, and non-eradicated. PCoA of all longitudinal samples showed no significant differences among the three clinical groups (**Figure 5A**). In the eradicated group, paired before-and-after samples demonstrated a clear and significant shift in the microbial community structure following treatment (*P* = 0.001) (**Figure 5B**). In the recurrent GC after eradication group, a significant difference between the before and after samples was also observed (*P* = 0.026), although the trajectories were heterogeneous in direction and magnitude across individuals (**Figure 5C**). In contrast, the non-eradicated group showed no significant change in microbial community structure over time, despite some inter-individual variability (**Figure 5D**).

Phylum-level stacked bar plots of the three groups showed that successful eradication was accompanied by a reduction in *H. pylori* abundance and a relative expansion of non-*Helicobacter* taxa, including Proteobacteria other than *H. pylori*, Firmicutes, Actinobacteria, and Bacteroidetes (**Figure 5E**). Individual-level phylum profiles further illustrated marked inter-individual heterogeneity after follow-up within each clinical group, including the eradicated (**Figure 5F**), recurrent GC after eradication (**Figure 5G**), and non-eradicated groups (**Figure 5H**).

### 3.6 Longitudinal microbial dynamics and taxa associated with *H. pylori* eradication status

**Figure 6:**
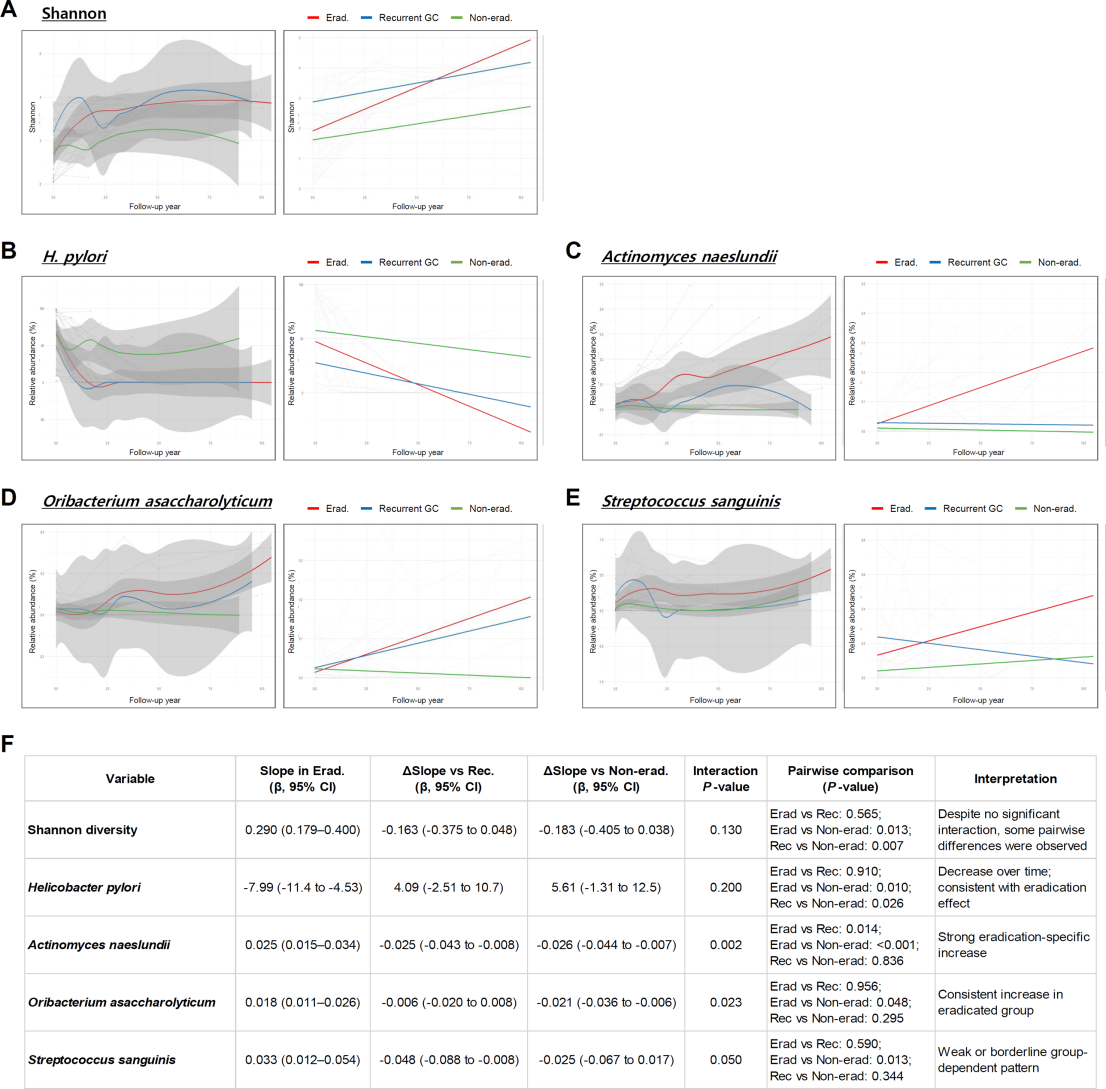
Longitudinal dynamics of microbial diversity and selected taxa based on linear mixed-effects models. **(A)** Temporal changes in Shannon diversity index across eradicated, recurrent GC, and non-eradicated groups. **(B)** Modeled abundance of *H. pylori*. **(C–E)** Modeled trajectories of selected taxa showing group-dependent changes, including *Actinomyces naeslundii*, *Oribacterium asaccharolyticum*, and *Streptococcus sanguinis*. **(F)** Summary of linear mixed-effects model results, including slopes, interaction effects, and pairwise comparisons. Lines represent estimated marginal means derived from linear mixed-effects models, and shaded areas indicate confidence intervals. Red, blue, and green lines correspond to eradicated, recurrent GC, and non-eradicated groups, respectively. LMM, linear mixed-effects model; GC, gastric cancer.

Longitudinal analysis using linear mixed-effects models revealed distinct temporal patterns in microbial diversity and taxonomic composition according to the eradication status (**Figure 6**). Alpha diversity, represented by the Shannon index, showed a gradual increase over time across all groups; however, no significant time-by-group interaction was observed (*P* = 0.130), indicating that overall diversity changes were not specific to the eradication status (**Figures 6A, F**). At the species level, *H. pylori* abundance decreased over time, consistent with the eradication effects, although the interaction was not statistically significant (*P* = 0.200) (**Figures 6B, F**). Among the taxa analyzed, *Actinomyces naeslundii* exhibited a significant time-by-group interaction (*P* = 0.002), with a marked increase exclusively in the eradicated group, whereas it remained relatively stable in the recurrent GC and non-eradicated groups (**Figures 6C, F**). This suggests that a selective increase is associated with successful eradication. *Oribacterium asaccharolyticum* also demonstrated a significant interaction (*P* = 0.023), with an increasing trend in the eradicated group compared with the non-eradicated group (**Figures 6D, F**). In contrast, *Streptococcus sanguinis* exhibited a borderline interaction effect (*P* = 0.050), suggesting a weaker and less consistent group-dependent pattern (**Figures 6E, F**). Overall, these findings indicate that, while global microbial diversity changes occur over time, species-level analyses reveal selective and *H. pylori* eradication-associated microbial shifts, with *A. naeslundii* emerging as the most robust *H. pylori* eradication-specific taxon.

### 3.7 Differentially abundant taxa associated with *H. pylori* eradication outcomes

**Figure 7:**
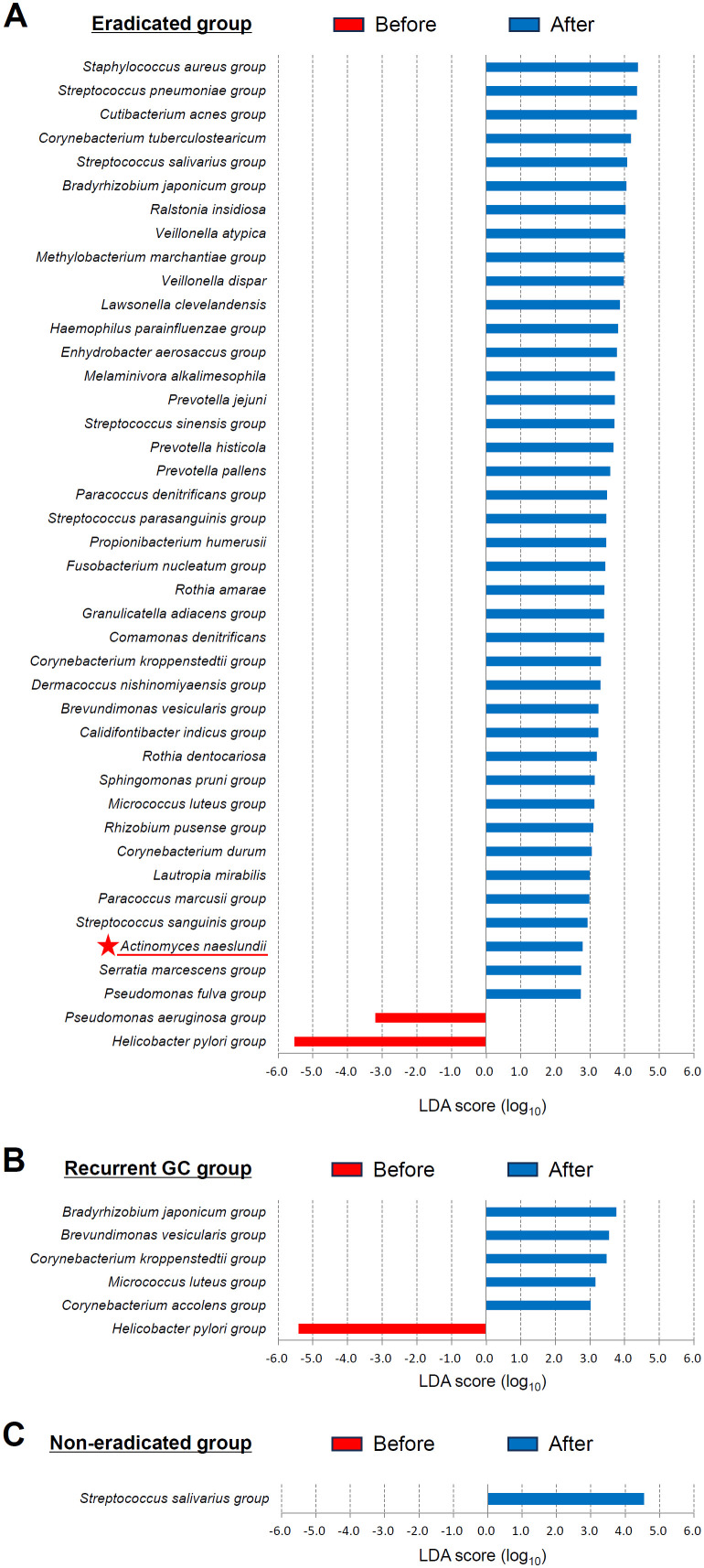
Differentially abundant taxa associated with eradication outcomes. **(A–C)** LEfSe analysis comparing baseline and follow-up samples in the eradicated **(A)**, recurrent GC after eradication **(B)**, and non-eradicated group **(C)**. Only taxa with defined species-level annotation and LDA scores above the predefined threshold (≥2.7-fold) are shown. Red bars indicate taxa enriched at baseline, and blue bars indicate taxa enriched at follow-up. LEfSe, linear discriminant analysis effect size; LDA, linear discriminant analysis; GC, gastric cancer.

To further characterize microbial changes before and after eradication, LEfSe analysis was performed for each clinical group (**Figure 7**). In the *H. pylori* eradicated group, a broad range of taxa was enriched at follow-up compared to baseline, whereas *H. pylori* and *Pseudomonas aeruginosa* were enriched at baseline, reflecting successful eradication (**Figure 7A**). At the follow-up, multiple taxa were enriched, including *Staphylococcus aureus*, *Streptococcus pneumoniae*, *Cutibacterium acnes*, *Corynebacterium tuberculostearicum*, *Streptococcus salivarius*, *Veillonella atypica*, *Prevotella jejuni*, *Fusobacterium nucleatum*, *Granulicatella adiacens*, and *Micrococcus luteus*. Notably, *Actinomyces naeslundii* was enriched at follow-up. Importantly, *A. naeslundii* was not identified as a discriminative taxon in comparisons based on *H. pylori* infection status (**Figures 2**-**4**) nor in subgroup analyses stratified by sex or histological subtype and was also not detected in longitudinal comparisons within the recurrent GC or non-eradicated groups. In contrast, the recurrent GC after eradication group showed an enrichment of *Bradyrhizobium japonicum*, *Brevundimonas vesicularis*, *Corynebacterium kroppenstedtii*, *Micrococcus luteus*, and *Corynebacterium accolens* at follow-up (**Figure 7B**). Similarly, the non-eradicated group exhibited minimal changes, with only *Streptococcus salivarius* being enriched at follow-up (**Figure 7C**).

### 3.8 Predicted functional shifts associated with *H. pylori* infection and its eradication status

**Figure 8:**
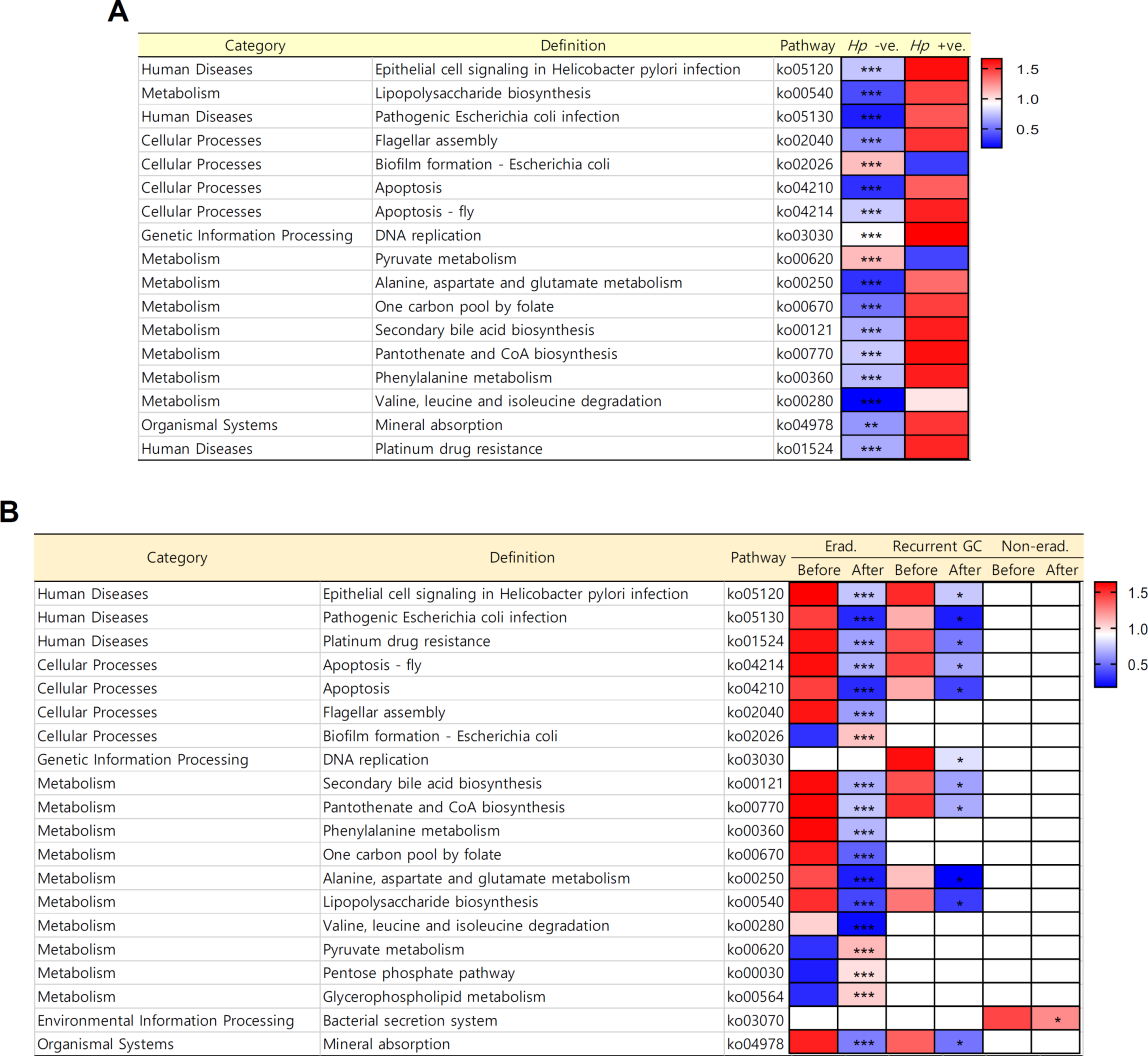
Predicted functional pathways based on KEGG annotations associated with *H. pylori* infection and eradication status. **(A)** Relative representation of predicted pathways between *H. pylori*–negative and *H. pylori*–positive samples **, *P* < 0.01; ***, *P* < 0.001 for two-group comparison. **(B)** Relative changes in predicted functional pathways between baseline and follow-up samples across eradicated, recurrent GC after eradication, and non-eradicated groups. Functional profiles were predicted from 16S rRNA gene sequencing data using KEGG-based pathway inference. *, *P* < 0.05; ***, *P* < 0.001 for comparison between baseline and follow-up in each group. KEGG, Kyoto Encyclopedia of Genes and Genomes.

Predicted functional profiling revealed consistent pathway-level differences according to *H. pylori* status at baseline (**Figure 8A**). *H. pylori*–negative samples showed lower relative representation of multiple pathways than *H. pylori*–positive samples, particularly those related to host– pathogen interactions and bacterial activity, including epithelial cell signaling in *H. pylori* infection, lipopolysaccharide biosynthesis, pathogenic *Escherichia coli* infection, flagellar assembly, apoptosis, and DNA replication, as well as several metabolic pathways.

Temporal comparisons between baseline and follow-up revealed distinct patterns of functional changes according to the eradication status (**Figure 8B**). In the eradicated group, pathways that were upregulated at baseline showed a consistent reduction at follow-up across multiple functional categories, including host interactions, cellular processes, and metabolism. In the recurrent GC after eradication group, similar directional changes were observed; however, these changes were less consistent and involved fewer pathways. In contrast, the non-eradicated group showed minimal longitudinal changes, with most pathways remaining stable between baseline and follow-up, indicating limited functional alterations over time.

## 4 Discussion

In this longitudinal cohort study, we characterized the gastric mucosa–associated microbiota in GC according to *H. pylori* infection status and *H. pylori* eradication outcomes. *H. pylori*–positive samples showed reduced diversity and dominance of *H. pylori*, whereas *H. pylori*–negative samples exhibited greater diversity with enrichment of non-*Helicobacter* taxa, including oral-associated bacteria such as *Streptococcus*, *Prevotella*, and *Neisseria*, suggesting potential oral–gastric microbial overlap or translocation under altered gastric conditions (Bik et al., 2006; Aviles-Jimenez et al., 2014; Kim et al., 2025). Several of these taxa possess urease-producing and nitrate-reducing capacities, indicating alternative metabolic pathways in the absence of *H. pylori*. Longitudinally, eradication led to heterogeneous microbial restructuring, whereas persistent infection maintained a stable *H. pylori*–dominated profile. Notably, *Actinomyces naeslundii* was uniquely enriched after eradication, suggesting its potential as a microbial signature of post-eradication ecological transitions, linking cross-sectional and temporal changes.

The reduced microbial diversity observed in *H. pylori*–positive samples reflects the organism’s strong ecological dominance in the gastric niche. *H. pylori* modulates gastric acidity, induces inflammation, and competitively excludes other microbial taxa, thereby shaping low-diversity microbial ecosystems (Cella et al., 2024; Jin et al., 2024). These effects are consistent with those of previous studies that demonstrated the dominant ecological role of *H. pylori* in shaping gastric microbial communities. In contrast, the absence of *H. pylori* permits colonization by a broader range of microorganisms, resulting in a more diverse but heterogeneous microbial community, as has been consistently reported in both GC and non-cancer cohorts (Bik et al., 2006; Aviles-Jimenez et al., 2014).

Following *H. pylori* eradication, we observed a shift toward increased microbial diversity and expansion of non-*Helicobacter* taxa, which is consistent with previous reports demonstrating partial microbial restructuring after *H. pylori* eradication (Li et al., 2017; Mao et al., 2021). However, these changes were highly variable across individuals, indicating that *H. pylori* eradication does not result in a uniform restoration of a “normal” microbiota but instead leads to divergent microbial trajectories depending on host and environmental factors, as suggested in previous microbiome studies (Noto and Peek, 2017). Our findings provide clinically relevant insights into gastric carcinogenesis. Persistent *H. pylori* infection was associated with a relatively stable microbial profile, suggesting ongoing exposure to a pro-inflammatory and carcinogenic microenvironment, whereas *H. pylori* eradication led to a shift toward a more diverse but heterogeneous microbial community, indicating only a partial restoration of microbial equilibrium (Park et al., 2019; Stewart et al., 2020).

Importantly, longitudinal modeling using LMM revealed that global diversity metrics alone did not capture *H. pylori* eradication-specific microbial dynamics. Although alpha diversity increased over time across all groups, no significant time-by-group interaction was observed, indicating that diversity changes were not specific to *H. pylori* eradication status. In contrast, species-level analyses have identified selective microbial shifts, highlighting the importance of longitudinal modeling and taxon-level resolution in microbiome research (Caporaso et al., 2011; Quince et al., 2017).

At the species level, *Actinomyces naeslundii* exhibited a significant time-by-group interaction, with a marked increase exclusively in the *H. pylori* eradicated group, whereas it remained relatively stable in both the recurrent and non-eradicated groups. *A. naeslundii* is a well-established oral commensal bacterium characterized by strong adhesion to mucosal surfaces and a robust biofilm-forming capacity, enabling its persistence in polymicrobial communities (Kolenbrander et al., 1993, 2006). These properties are primarily mediated by fimbria-dependent adhesion mechanisms (Ruhl et al., 2004). In addition, oral microbial communities, including Actinomyces species, have been implicated in nitrate reduction pathways (Hezel and Weitzberg, 2015), suggesting their potential role in microbial adaptation under altered ecological conditions. This group-specific pattern suggests that its enrichment is not merely a passive consequence of microbial recovery, but may reflect context-dependent microbial expansion following the removal of *H. pylori*. Given its role as an early colonizer in biofilm formation, *A. naeslundii* may be involved in the early stage microbial community reorganization post-*H. pylori* eradication under normal gastric conditions. Therefore, its emergence may represent a candidate indicator of microbial ecological transition rather than a static disease-associated taxon.

This pattern was consistent with the broader enrichment of oral-associated taxa observed in *H. pylori*–negative and post-eradication conditions. In addition, *H. pylori*–negative samples were enriched with diverse non-*Helicobacter* taxa, including organisms commonly associated with the oral microbiome, such as *Streptococcus*, *Prevotella*, and *Neisseria*. Although these taxa are typically commensal in their native oral niche, their presence in the gastric environment may reflect an ecological displacement under altered physiological conditions. These findings are consistent with those of previous studies that demonstrated increased representation of oral-associated taxa in the gastric mucosa under conditions of reduced gastric acidity or mucosal barrier disruption (Bik et al., 2006; Aviles-Jimenez et al., 2014; Kim et al., 2025). Therefore, the gastric environment in *H. pylori*–negative or post-eradication states may facilitate colonization by orally derived taxa, potentially through reduced colonization resistance, altered gastric acidity, and changes in local ecological niches.

Notably, previous longitudinal studies in non-cancer populations have demonstrated that gastric microbiota in *H. pylori*–uninfected individuals exhibit distinct compositional patterns and does not necessarily converge toward a uniform “healthy” state but instead follows heterogeneous and context-dependent ecological trajectories (Shin et al., 2020). These findings support our observations in patients with GC, suggesting that post-*H. pylori* eradication microbial restructuring reflects context-dependent ecological dynamics rather than a simple return to a normal microbiome state.

Several of these taxa, including *Staphylococcus aureus* and *Corynebacterium tuberculostearicum*, possessed urease-producing and nitrate-reducing capabilities, which may contribute to gastric carcinogenesis through multiple biochemical mechanisms. Urease activity may promote ammonia production and local pH alteration, contributing to epithelial injury and chronic inflammation (Eaton et al., 1991; Mobley et al., 1991), and carcinogenic N-nitroso compound formation under hypochlorhydric conditions (Keszei et al., 2013; Seyyedsalehi et al., 2023). These processes are supported by the microbial nitrosation pathways previously described in gastric and oral microbiomes (Jo et al., 2016; Qu et al., 2016). These findings support a mechanistic model in which non-*Helicobacter* microbial communities contribute to gastric carcinogenesis through biochemical pathways distinct from those driven by *H. pylori*, particularly under specific ecological conditions. Previously we reported that the changes of prevalence of *H. pylori*-positive GC decreased from 93.4% (436/467) in 2003~2007, to 88.5% (500/565) in 2008~2012, and to 82.1% (160/195) in 2013~2018, respectively (*P* < 0.001) (Lee et al., 2019). This report supports the potential non-*Helicobacter* microbial contributions to gastric carcinogenesis. Although we attempted to identify specific bacterial taxa consistently associated with *H. pylori*-negative GC, no single dominant microbial species was reproducibly identified across cases. Therefore, we adopted a functional characteristic–based approach focusing on urease-producing and nitrate-reducing potential as biologically plausible microbial features associated with gastric carcinogenesis, consistent with previous studies of *H. pylori*-negative gastric microbiota (Sohn et al., 2017). Taken together, these results suggest that the post-eradication gastric microbiome does not converge toward a single “normal” state but instead exhibits heterogeneous microbial configurations with distinct ecological implications.

However, the composition of these non-*Helicobacter* taxa varied substantially across individuals and clinical groups, which is consistent with previous studies reporting high inter-individual heterogeneity in the gastric microbiome of patients with GC (Zhang et al., 2024). This heterogeneity suggests that *H. pylori*–negative GC does not follow a single canonical microbiome pattern but instead may represent multiple distinct ecological states (Cho and Blaser, 2012). Similarly, in our longitudinal analysis, the taxa enriched in the recurrent GC group differed from those observed in the eradicated group, further supporting the concept of divergent microbial trajectories with potentially distinct biological implications.

Consistent with this variability, previous studies have reported enrichment of diverse microbial taxa in GC, including *Prevotella*, *Veillonella*, *Parvimonas*, *Peptostreptococcus*, *Actinomyces*, *Rothia*, *Fusobacterium*, and *Streptococcus anginosus*, although these findings have been highly heterogeneous across studies and populations (Liu et al., 2022a, 2022; Zeng et al., 2024). Consistent with this, our longitudinal analysis identified additional taxa that were enriched in the recurrent GC group after *H. pylori* eradication, suggesting that multiple microbial configurations may be associated with disease progression.

Stratified analyses further indicated that host- and tumor-related factors modulate the gastric microbiome in a context-dependent manner. Although *H. pylori* status exerted the strongest influence, sex-specific differences were observed, with females exhibiting more pronounced changes in microbial diversity and composition. Experimental evidence supports these observations, demonstrating sex-dependent immune and microbial responses to *H. pylori* infection, which are potentially mediated by estrogen (Ohtani et al., 2007; Peng et al., 2025). Experimental models further suggest that estrogen may modulate microbial composition and inflammatory responses, contributing to higher microbial diversity and reduced inflammatory burden in females compared to males. In addition, microbial patterns varied according to GC subtype, consistent with studies showing that the histological phenotype is associated with microbial diversity and composition (Sarhadi et al., 2021; Booth et al., 2025). Notably, intestinal-type GC has been associated with higher microbial diversity and abundance than diffuse-type GC, suggesting that tumor biology itself may shape the microbial landscape (Sarhadi et al., 2021; Booth et al., 2025). These findings support a hierarchical model in which *H. pylori* acts as the primary ecological driver, whereas host and tumor factors function as secondary modifiers.

Functional predictions further support these ecological differences. *H. pylori*–positive samples were enriched in pathways related to epithelial cell signaling, lipopolysaccharide biosynthesis, bacterial pathogenicity, and cellular processes, such as apoptosis and flagellar assembly, consistent with previous functional analyses of *H. pylori*–associated microbiota (Wang et al., 2020; Huang et al., 2024). In contrast, these pathways were reduced following *H. pylori* eradication, suggesting the attenuation of host–pathogen interaction signals. Although these findings are based on 16S rRNA–derived functional inferences and require validation using metagenomic or metabolomic approaches, they provide preliminary insights into the potential functional shifts associated with microbial restructuring.

This study has several strengths. The use of gastric mucosa–associated samples provides direct insights into the local microbial environment at the disease site. The longitudinal design enables the assessment of temporal microbial dynamics that are not apparent in cross-sectional analyses, particularly in the context of *H. pylori* eradication, where microbial changes are heterogeneous and require longitudinal modeling to identify group-specific trajectories. In addition, stratified analyses by sex and histologic subtype provided a more refined understanding of host–microbiome interactions in GC.

However, this study had several limitations. First, the sample size, particularly within the recurrent GC and other longitudinal subgroups, was relatively small, which may limit the statistical power and generalizability of the findings. Therefore, subgroup-specific findings should be interpreted cautiously and considered exploratory. Longitudinal gastric microbiome studies involving repeated endoscopic biopsy sampling and long-term follow-up after *H. pylori* eradication are inherently difficult to perform in clinical practice because repeated surveillance sampling requires continuous patient participation and consent. In addition, repeated confirmation of *H. pylori* infection status during long-term follow-up is not routinely available in many patients. Furthermore, recurrent GC after successful endoscopic treatment and *H. pylori* eradication is relatively uncommon, with reported recurrence rates generally below approximately 7.5% (Yoon et al., 2016), making it particularly difficult to establish a large recurrent GC longitudinal cohort. Second, the functional prediction analysis was based on 16S rRNA gene sequencing and therefore reflects inferred rather than directly measured microbial functions. Accordingly, the predicted functional pathways should be interpreted cautiously (Quince et al., 2017). Future studies using shotgun metagenomic or metatranscriptomic approaches may help validate the functional relevance of these microbial alterations. Third, sampling was limited to specific gastric mucosal site, corpus, which may not fully capture spatial heterogeneity.

In conclusion, our findings demonstrate that *H. pylori* functions as a primary ecological driver of the gastric microbiome, shaping a low-diversity, *H. pylori*–dominated state, whereas *H. pylori* eradication induces heterogeneous and taxon-specific microbial restructuring rather than uniform restoration. The enrichment of non-*Helicobacter* taxa with urease- and nitrate-reducing potential suggests that alternative microbial pathways may contribute to gastric carcinogenesis. The identification of *Actinomyces naeslundii* as a selectively enriched taxon following *H. pylori* eradication highlights its potential as a biomarker for post-eradication ecological transitions. Together, these findings underscore the complexity and heterogeneity of gastric microbial ecosystems and support the need for future multi-omics studies to clarify their functional and clinical implications.

## Data Availability

The raw 16S rRNA gene sequencing data generated in this study have been deposited in the National Center for Biotechnology Information Sequence Read Archive under the BioProject accession number PRJNA1447187 and are publicly available at http://www.ncbi.nlm.nih.gov/bioproject/1447187. Additional data supporting the findings of this study are provided in the **Supplementary Material.**.
